# Circulating *miR‐542‐3p* as a Prognostic Marker for Hepatocellular Carcinoma: A Systematic Review and Meta‐Analysis

**DOI:** 10.1111/jcmm.70748

**Published:** 2025-07-27

**Authors:** Ranjith Balakrishnan, Rajasekaran Subbarayan, Maheshkumar Kuppusamy, Rupendra Shrestha, Arunkumar Radhakrishnan, Ankush Chauhan

**Affiliations:** ^1^ Centre for Advanced Biotherapeutics and Regenerative Medicine, Faculty of Research Chettinad Hospital and Research Institute, Chettinad Academy of Research and Education Kelambakkam Tamil Nadu India; ^2^ Centre for Herbal Pharmacology and Environmental Sustainability Chettinad Hospital and Research Institute, Chettinad Academy of Research and Education Kelambakkam Tamil Nadu India; ^3^ Department of Physiology & Biochemistry Government Yoga and Naturopathy Medical College and Hospital Chennai Tamil Nadu India; ^4^ Department of Natural and Applied Sciences Nexus Institute of Research and Innovation (NIRI) Lalitpur Nepal; ^5^ Department of Pharmacology Chettinad Hospital and Research Institute, Chettinad Academy of Research and Education Kelambakkam Tamil Nadu India

**Keywords:** cancer progression, hepatocellular carcinoma, liver, *miR‐542‐3p*, prognostic biomarker

## Abstract

Hepatocellular carcinoma (HCC) is a leading cause of cancer‐related mortality worldwide, particularly in Asia. Despite therapeutic advancements, the prognosis of HCC remains poor. MicroRNAs have emerged as potential biomarkers for HCC prognosis and therapeutic response. This systematic review and meta‐analysis examined the association between circulating *miR‐542‐3p* levels and HCC progression. Seven studies on HCC and *miR‐542‐3p* were selected for the meta‐analysis. *miR‐542‐3p* showed significant diagnostic potential for HCC prognosis and therapeutic evaluation. Two independent researchers performed data extraction and used the Quality Assessment of Diagnostic Accuracy Studies (QUADAS‐2) method to assess the quality and risk of bias of the included studies. After the meta‐analysis, human miRNA pattern analysis was conducted using the miRBase database, and the expression of *miR‐542‐3p* was confirmed based on the results obtained from the human miRNA profile in the tissue atlas database. *miR‐542‐3p* has been shown to have significant diagnostic potential for HCC prognosis and therapeutic evaluation. The pooled sensitivity was 0.79 (95% CI, 0.75–0.83), while the pooled specificity was 0.34 (95% CI, 0.29–0.40). The diagnostic odds ratio was 7.2, with an AUC of 0.806, indicating moderate diagnostic accuracy. Network analysis in miRBase links *miR‐542‐3p* to liver function, and its location on the X chromosome allows its expression in both sexes, making it widely applicable for the diagnosis of HCC. Thus, *miR‐542‐3p* has potential as a prognostic biomarker for HCC, with prospects for integration into therapeutic strategies. Future studies should explore the combination of this with targeted therapies to improve patient outcomes.

## 1 Introduction

Various factors contribute to the development of liver cancer, including excessive alcohol consumption, Hepatitis B or C virus infection, and exposure to aflatoxin [1]. Hepatocellular carcinoma (HCC) is a significant contributor to cancer‐related mortality worldwide. According to recent statistics, the global incidence of liver cancer is expected to reach approximately 9,05,677 cases by 2023, with an estimated annual mortality rate of approximately 830,180. Asia accounts for approximately 72% of cases, whereas Europe accounts for approximately 10% of cases [2, 3]. The therapeutic approaches for HCC rely on surgical intervention, radiotherapy, and chemotherapy. However, the prognosis of these patients remains poor, particularly in those who develop resistance to chemotherapy [4]. Despite notable advancements in therapeutic approaches, including hepatectomy and targeted therapy, the prognosis of patients with HCC remains unfavourable owing to the frequent occurrence of intrahepatic and distal metastases [5]. MicroRNAs (miRNAs) play a crucial role in controlling the translation and degradation of target mRNA. They achieve this by binding to specific sequences within the 30‐untranslated region (UTR) of mRNA [6]. Over the past few years, extensive research has highlighted the significant impact of miRNAs on a wide range of biological processes, including proliferation, apoptosis, and metastasis. Additionally, these miRNAs have shown potential as biomarkers for predicting the therapeutic response and prognosis of patients with HCC [7]. Recent studies have revealed the involvement of *miR‐542‐3p* in the progression of various cancers [8, 9].

A study by Cui et al. revealed a significant discovery regarding the inhibition of invasion and metastasis in melanoma. This study highlights the crucial role of *miR‐542‐3p* in this process. This is achieved by specifically targeting the serine/threonine protein kinase PIM1 [10]. A study conducted by Wu et al. in the field of HCC revealed an interesting discovery: the inhibitory effect of *miR‐542‐3p* on the development of HCC. Inhibition occurs by targeting the FZD7/Wnt signalling pathway, whereas downregulation of *miR‐542‐3p* promotes cancer metastasis by activating the TGF‐β/Smad signalling pathway in HCC [11]. However, the exact effect of *miR‐542‐3p* on HCC spread remains unclear. We performed a meta‐analysis to explore the prognostic effects of *miR‐542‐3p* expression on HCC progression. Specifically, we used different software and databases to conduct a meta‐analysis to determine the correlation between *miR‐542‐3p* and HCC.

## 2 Methods

This systematic review and meta‐analysis was reported in accordance with the PRISMA (Preferred Reporting Items for Systematic Reviews and Meta‐Analyses) and AMSTAR (Assessing the Methodological Quality of Systematic Reviews) guidelines [12, 13]. The study protocol was prospectively registered in the International Prospective Register of Systematic Reviews (PROSPERO: CRD42024593014).

### 2.1 Inclusion and Exclusion Criteria

This study aimed to investigate the effects of *miR‐542‐3p* in patients diagnosed with HCC. The case group comprised individuals diagnosed with HCC and with a history of alcohol consumption, whereas the control group included healthy individuals or patients without a history of alcohol consumption. We conducted a comprehensive literature review and gathered a set of diagnostic data, including true positive, false positive, true negative, and false negative results. In accordance with our inclusion criteria, original research articles presenting quantitative data relevant to the research question, availability of full text in English, clearly defined study design and methodology, and sufficient statistical information to extract or compute effect estimates, we excluded studies that did not meet these standards. Specifically, the exclusion criteria encompassed animal studies, narrative reviews, study protocols, unpublished data, editorials, clinical case reports, commentaries, abstracts, lectures, non‐primary research articles, dissertations, and conference proceedings, as they lacked the methodological rigour or data required for meta‐analytic synthesis. These sources were deemed irrelevant to the focus of the study. We limited our review to preprints and articles published in English without restrictions on geographical location or research settings.

### 2.2 Search Strategy and Screening

We conducted a comprehensive literature search of multiple scientific databases, including PubMed, Web of Science, Scopus, and EMBASE. We focused on publications in English between 2010 and 2024, specifically studies on the potential therapeutic and prognostic significance of *miR‐542‐3p* in HCC. We used a combination of keywords, MeSH terms, controlled vocabulary, synonyms, and additional search terms pertinent to *miR‐542‐3p*, liver, and HCC. We carefully checked for duplicate entries and performed screening and extraction. Two independent researchers screened the titles and abstracts and excluded irrelevant studies. Full‐text articles were then reviewed for final inclusion based on their relevance. In cases of disagreement, a third senior researcher resolved the discrepancies.

### 2.3 Data Extraction

Two independent researchers extracted data from the seven selected studies. The extracted data included the lead author's name, country where the study was conducted, duration of the research, publication year, and diagnostic statistics such as true positive, false positive, false negative, and true negative values for the index tests. To ensure precision, data extraction was performed twice (Table S1).

### 2.4 Risk of Bias Assessment

We used the Quality Assessment of Diagnostic Accuracy Studies (QUADAS‐2) tool to assess the potential bias and relevance of proficiency studies. We created a methodological integrity graph using Review Manager (RevMan 5.4).

### 2.5 Statistical Analysis

MetaDiSc 1.4 was used to calculate the sensitivity and specificity and to generate the SROC curve for *miR‐542‐3p* in diagnosing HCC from the collected data. Python 3.12.5 was used for further validation and statistical analysis (quartiles 1 and 3 and *miR‐542‐3p* log2 expression). GraphPad Prism 10.3.0 was used to perform the Student's *t*‐test to calculate the significance (***p < 0.0001) of two variables with the generation of the ROC curve, and sample distribution was analysed using Python 3.12.5.

## 3 Results

### 3.1 Study Inclusion and Quality Assessment

**FIGURE 1:**
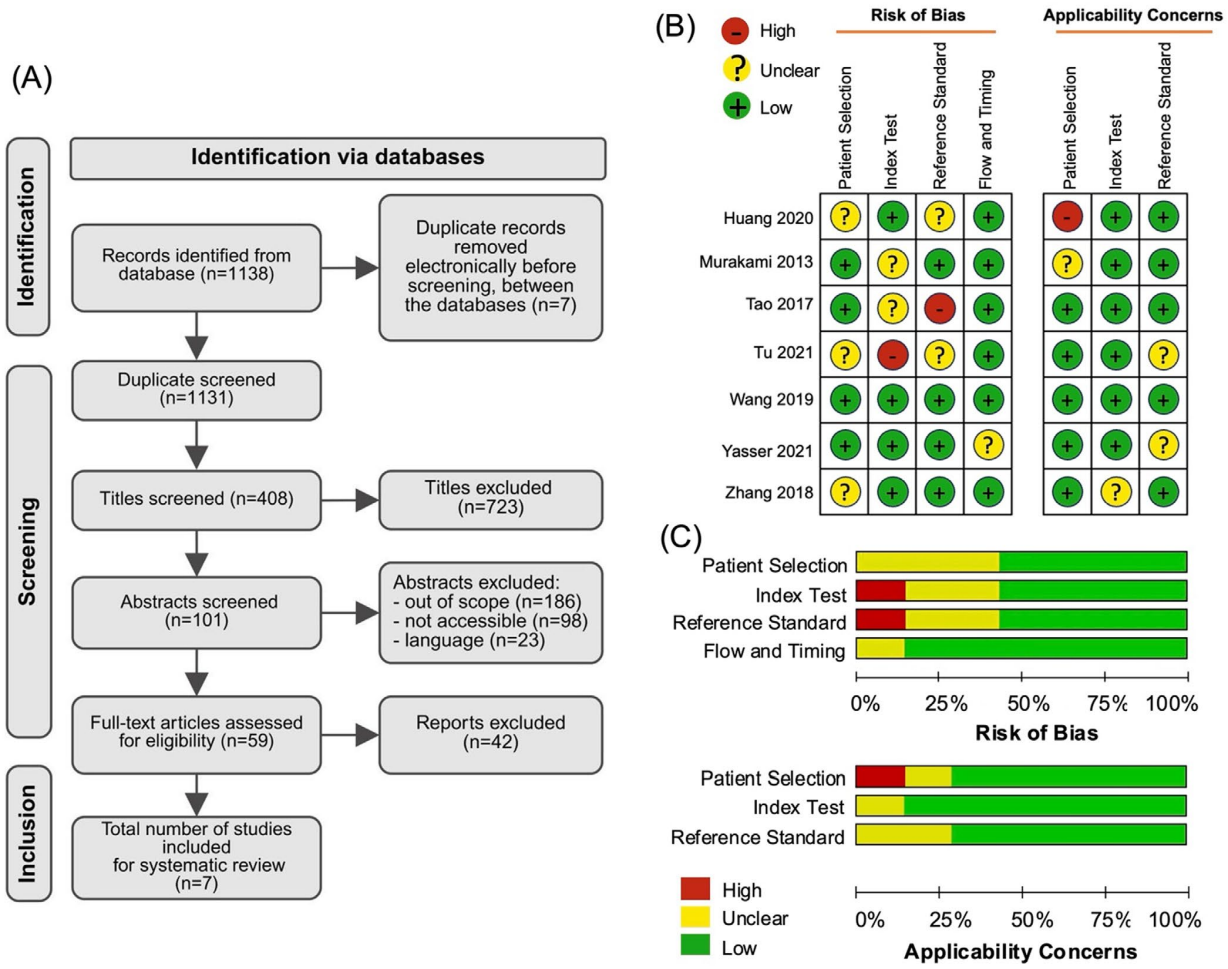
(A) Study selection shows the identification, screening, and inclusion criteria based on PRISMA standards. (B, C) Methodological quality and summary of bias risk assessed based on the QUADAS‐2 guidelines.

A study flow diagram based on the PRISMA standards for the recognition and preference of studies is presented in Figure 1A. From an initial pool of 1138 identified records, seven duplicates were removed, resulting in 1131 records for screening. Following this screening process, 408 titles were selected for further review. A total of 307 titles were subsequently excluded due to irrelevance, such as inaccessibility (*n* = 98), language barriers (*n* = 23), or being outside the scope of the study (*n* = 186), leaving 101 abstracts to be assessed for eligibility. Subsequently, 59 full‐text articles were evaluated based on the predefined inclusion criteria, relevance to the research question, English language, accessibility, and sufficient methodological detail. Of these, 42 were excluded for not meeting the inclusion criteria of the study. Ultimately, seven studies that satisfied the predefined criteria were included in this systematic review. The included studies were evaluated for risk of bias and applicability concerns across four domains: patient selection, index test, reference standard, and flow and timing. Risk levels are represented by colour‐coded circles: red (high), yellow (unclear), and green (low). Most studies showed low bias (green circles), although some exhibited unclear risk in patient selection and high bias in the index test and flow and timing (yellow and red circles, respectively) (Figure 1B). Furthermore, the pooled results also indicated a low risk of bias across most domains; however, concerns were noted in the patient selection and index test domains of the studies. A study by Huang et al. [4] was identified as having a potential risk of bias in patient selection; however, the overall methodological quality remained acceptable for inclusion in the analysis (Figure 1C). This analysis underscored the quality of the included studies and identified areas of concern, particularly regarding patient selection and index test accuracy.

### 3.2 Pooled Analysis of Theragnostic Accuracy of *miR‐542‐3p*

**FIGURE 2:**
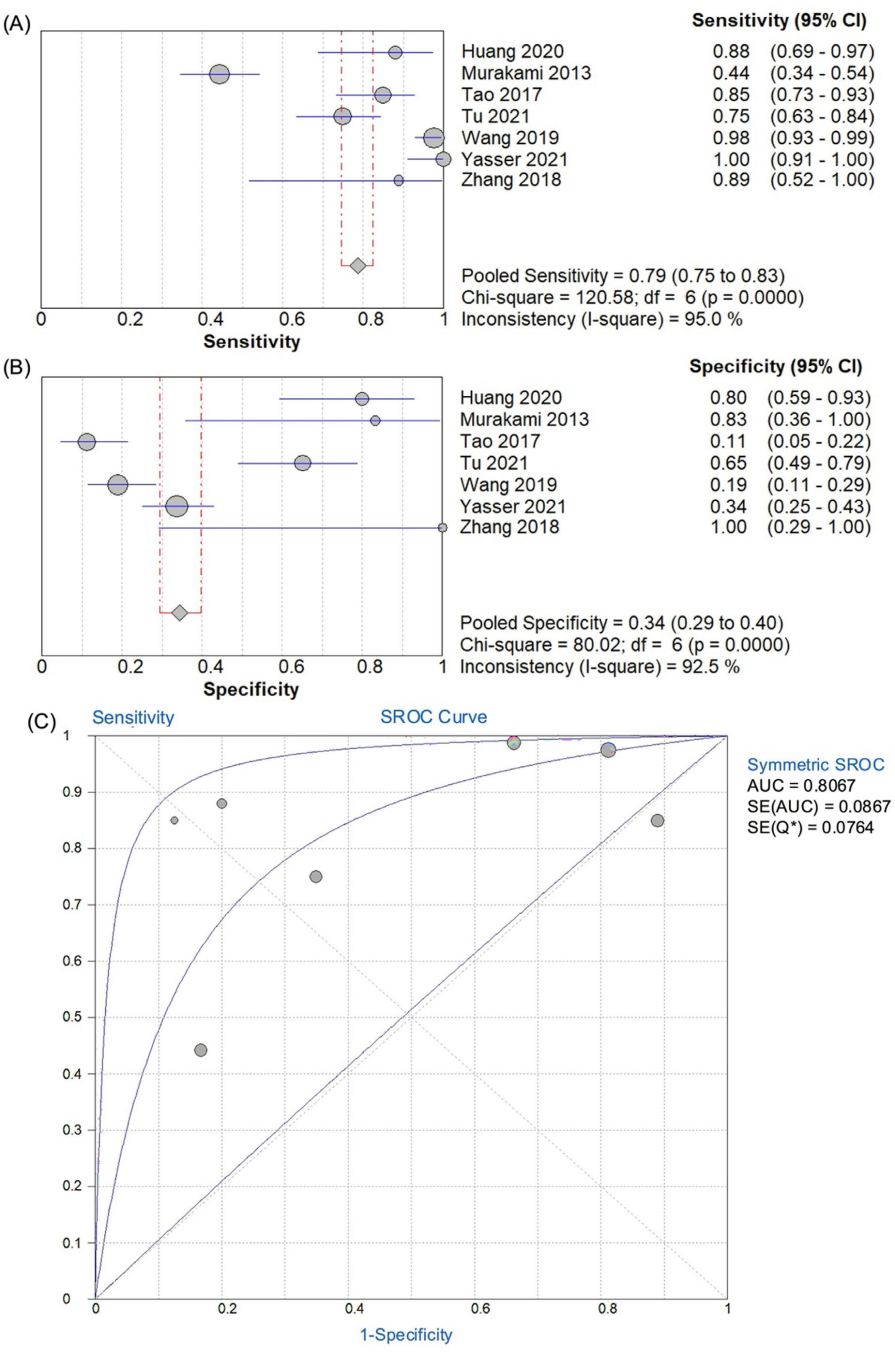
(A, B) Forest plot of summary sensitivity and specificity of circulating *miR‐542‐3p* levels for HCC diagnosis. (C) Symmetric summary receiver operating characteristic (SROC) threshold of *miR‐542‐3p* in HCC prognosis.

Seven studies that fulfilled the inclusion criteria were selected for the meta‐analysis (Table S1). Seven articles comprising 154 controls and 622 cases were analysed for the study. The pooled analysis of *miR‐542‐3p* for diagnosing HCC showed promising results, with a pooled sensitivity of 0.79 (95% CI: 0.75–0.83), suggesting good accuracy in detecting true positives across studies. However, significant heterogeneity (χ^2^ = 120.58, *I* ^2^ = 95%) indicated that factors such as variations in the study design or population characteristics may influence sensitivity. Sensitivity values ranged widely from 0.44 to 1.00, further highlighting these inconsistencies (Figure 2A). The specificity values ranged from 0.11 to 1.00, with a pooled specificity of 0.34 (95% CI: 0.29–0.40), which is relatively low. This makes *miR‐542‐3p* less reliable for excluding non‐HCC cases. The high heterogeneity observed in specificity (χ^2^ = 80.02, *I* ^2^ = 92.5%) suggests challenges in standardising *miR‐542‐3p* as a robust diagnostic tool to exclude false positives (Figure 2B). Additional metrics, such as a positive likelihood ratio (PLR) of 1.5 (95% CI, 1.14–2.03) and a negative likelihood ratio (NLR) of 0.3 (95% CI, 0.15–0.66), indicate that *miR‐542‐3p* may be more useful for confirming HCC when a positive result is obtained rather than ruling out the disease. The diagnostic odds ratio (DOR) of 7.2 (95% CI, 2.31–22.48) supports the potential diagnostic value of *miR‐542‐3p*, although with considerable variability between studies, as shown in (Figure S1). The pooled analysis indicated a clear association between *miR‐542‐3p* and HCC, as demonstrated by the calculated sensitivity and specificity of the data (Table 1). Overall, *miR‐542‐3p* is a potential diagnostic biomarker for HCC; however, its clinical utility is limited by its low specificity and high heterogeneity. Further well‐designed large‐scale studies are required to address these limitations of the current study.

**TABLE 1:** Results of subgroup analysis.

Ref.	Case	Control	TP	FP	FN	TN	Sensitivity (95% CI)	Specificity (95% CI)	PLR (95% CI)	NLR (95% CI)	DOR (95% CI)
Huang et al. [4]	25	25	22	5	3	20	0.88 [0.69, 0.97]	0.80 [0.59, 0.93]	4.4 [1.98, 9.76]	0.15 [0.05, 0.44]	29.33 [6.20, 138.78]
Murakami et al. [14]	104	6	46	1	58	5	0.44 [0.34, 0.54]	0.83 [0.36, 1.00]	2.6 [0.43, 16.09]	0.66 [0.45, 0.99]	3.96 [0.44, 35.13]
Tao et al. [15]	107	16	51	56	9	7	0.85 [0.73, 0.93]	0.11 [0.05, 0.22]	0.95 [0.83, 1.09]	1.35 [0.53, 3.39]	0.70 [0.24, 2.04]
Tu et al. [16]	69	46	54	15	18	28	0.75 [0.63, 0.84]	0.65 [0.49, 0.79]	2.1 [1.39, 3.30]	0.38 [0.24, 0.60]	5.60 [2.45, 12.75]
Wang et al. [17]	193	20	120	73	3	17	0.98 [0.93, 0.99]	0.19 [0.11, 0.29]	1.2 [1.08, 1.33]	0.12 [0.03, 0.42]	9.31 [2.63, 32.88]
Yasser et al. [18]	115	38	40	75	0	38	1.00 [0.91, 1.00]	0.34 [0.25, 0.43]	1.4 [1.30, 1.70]	0.03 [0.002, 0.57]	41.30 [2.47, 690.03]
Zhang et al. [1]	9	3	8	0	1	3	0.89 [0.52, 1.00]	1.00 [0.29, 1.00]	6.8 [0.50, 92.08]	0.17 [0.03, 0.78]	39.66 [1.27, 1229.9]

Abbreviations: CI, confidence interval; DOR, diagnostic odds ratio; FN, false negative; FP, false positive; NLR, negative likelihood ratio; PLR, positive likelihood ratio; TN, true negative; TP, true positive.

### 3.3 Summary Receiver Operating Characteristic Curve (SROC) Analysis

SROC analysis of *miR‐542‐3p* highlighted its potential as a diagnostic marker of HCC. With an Area Under the Curve (AUC) of 0.806, *miR‐542‐3p* demonstrated a strong diagnostic capability (Figure 2C). This suggests that the biomarker offers good accuracy in distinguishing HCC from non‐cancerous controls. A cutoff value of 0.806 indicates a biomarker with balanced sensitivity and specificity, making *miR‐542‐3p* a promising candidate for further development as a diagnostic tool. However, the steep initial rise in the curve reflects high sensitivity but low specificity (Figure 2C). High sensitivity ensures that *miR‐542‐3p* can effectively detect true‐positive cases, whereas its specificity ensures a lower rate of false positives (Figure 2A,B). Variability in specificity values among different studies, as noted in prior analyses, suggests the need for standardised testing protocols to optimise *miR‐542‐3p* performance across various clinical settings. Overall, *miR‐542‐3p* shows significant potential as a noninvasive diagnostic biomarker for HCC; however, further validation through large‐scale studies is required to standardise its application. This evidence supports its utility; however, further research is necessary to fully harness its diagnostic value.

### 3.4 Data Confirmation Using the miRBase Database

Human miRNA pattern analysis was performed using the miRBase database (http://www.mirbase.org/). The expression of *miR‐542‐3p* was confirmed based on the results obtained from the human miRNA profile in the Tissue Atlas database (https://ccb‐web.cs.uni‐saarland.de/tissueatlas). Python 3.12.5 was used to generate plots using the database information.

**FIGURE 3:**
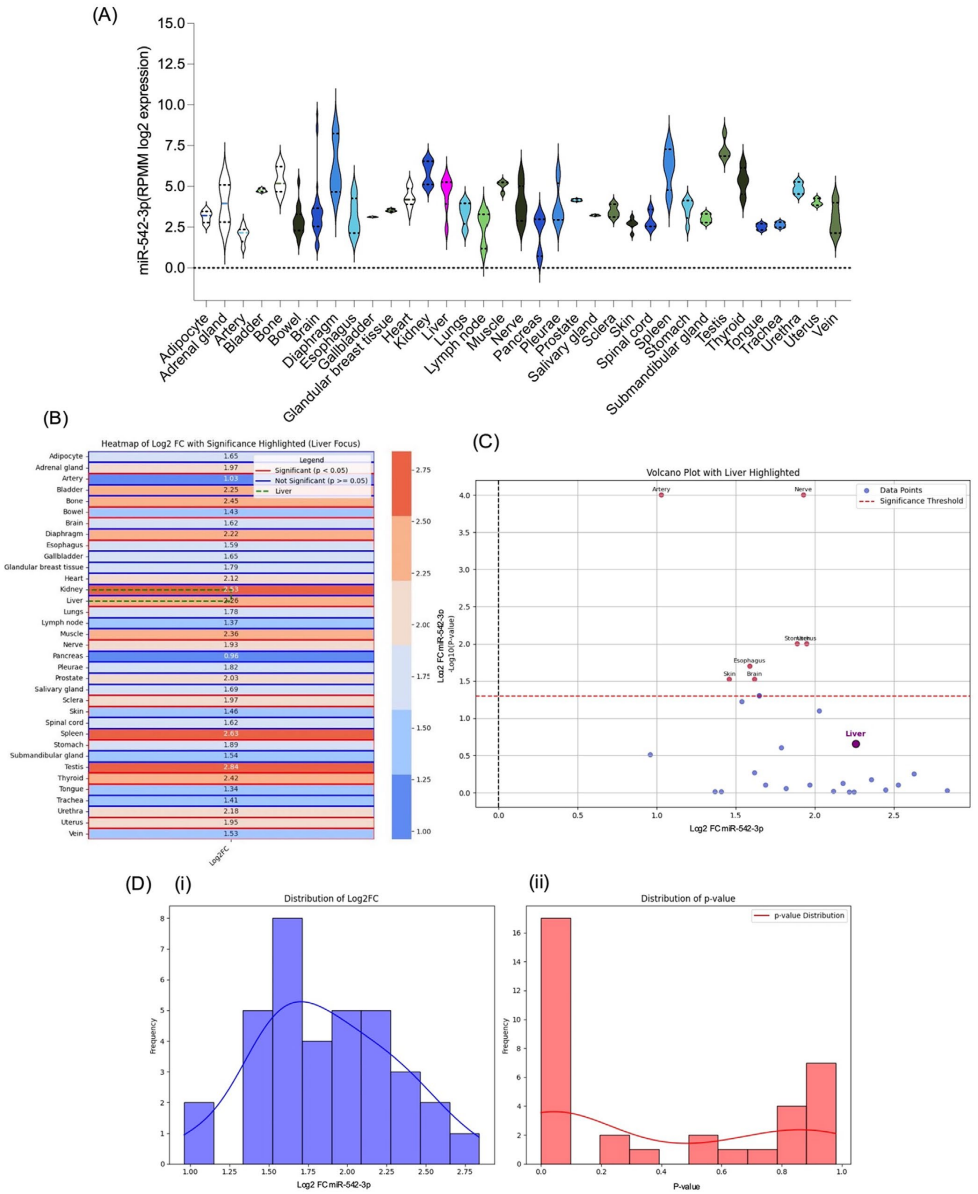
(A) Depicts a violin plot showing the range, density, and distribution of *miR‐542‐3p* expression across different tissues (http://www.mirbase.org/). (B) Heatmap illustrating the log2 fold‐change (FC) in *miR‐542‐3p* expression in various organs. (C) Volcano plot displaying the Log2 fold change versus −log10 (*p*‐value) for various tissues, with the liver specifically highlighted in purple. (D‐i) shows the distribution of Log2 fold change (Log2FC) across the dataset, indicating that the most observed changes were moderate in magnitude. (D‐ii) displays the distribution of *p*‐values, suggesting numerous statistically significant changes within the dataset. The red line in the right panel represents the smoothed density curve of the *p*‐value distribution, highlighting the frequency of lower *p*‐values compared to higher ones.

Distribution of log2 expression levels of *miR‐542‐3p* in various human tissues (Figure 3A). Among all examined tissues, the liver showed higher expression levels in terms of central tendency and distribution spread, indicating robust tissue‐specific enrichment of miR‐542‐3p. Notably, the liver exhibited a markedly higher expression than other tissues (Figure 3A). This finding suggests that *miR‐542‐3p* may serve as a potential liver‐specific biomarker crucial for understanding its role in HCC. Furthermore, the log2 fold change (FC) for *miR‐542‐3p* expression across various tissues highlighted the liver, with a log2 FC of 2.26, indicating significant overexpression. Other tissues, such as the oesophagus and stomach, also showed notable expression, but to a lesser extent (Figure 3B). This finding emphasises the crucial role of the liver in *miR‐542‐3p* regulation, which may be associated with liver‐specific processes. Additionally, the volcano plot highlights the liver tissue, showing the relationship between log2 FC and −log10 *p*‐values. It revealed a high log2 FC and low *p*‐value for the liver, indicating significant upregulation of *miR‐542‐3p* expression in the liver tissue compared to other tissues, such as the arteries, nerves, stomach, and oesophagus (Figure 3C). Furthermore, the log2 FC distribution for *miR‐542‐3p* across tissues mostly ranged between 1.0 and 2.75, indicating a moderate change in expression (Figure 3D‐i). However, many tissues exhibited a skewed *p*‐value distribution towards lower values, underscoring the statistical significance of the differences in *miR‐542‐3p* expression (Figure 3D‐ii). Network analysis of the interaction between *miR‐542‐3p* and its association with the liver. The red node represents *miR‐542‐3p*, and the blue nodes represent the liver. The edges (lines) connecting these nodes indicate possible biological interactions, such as gene regulation or functional linkage (Figure S2‐i). An enrichment plot shows the over‐representation of *miR‐542‐3p* in Chromosome X (miRBase database http://www.mirbase.org/), indicating that it can be expressed in both males and females (Figure S2‐ii).

## 4 Discussion

This study introduces a therapeutic approach for HCC prognosis by enhancing the expression of the newly identified target, *miR‐542‐3p*. miRNAs significantly impact the development and progression of various cancers, including HCC [19]. miRNAs have great potential as prognostic biomarkers and therapeutic targets in HCC [20]. Exosome‐derived miRNAs have been shown to effectively block cancer cell proliferation, and exosomes derived from TAMs contain miR‐142‐3p, which inhibits tumour growth and action by targeting RAC1 [21, 22]. Additionally, *miR‐542‐3p* has been identified as a newly discovered miRNA associated with cancer, and a potential gene that may help suppress the growth of malignancies in multiple forms [8]. Furthermore, the absence of *miR‐542‐3p* promotes the activation of IGFBP‐1 in human endometrial stromal cells undergoing decidualisation [23]. However, to our knowledge, no meta‐analysis has been performed on *miR‐542‐3p* to demonstrate its importance in alcohol‐induced liver cirrhosis. Therefore, we conducted a comprehensive meta‐analysis, registered under PROSPERO (*CRD42024593014*), to determine the quantitative association between *miR‐542‐3p* expression and HCC development. The chromosomal position and intersection size of *miR‐542‐3p* were acquired from the‘miRBase’ database to determine its exact location of *miR‐542‐3p*. Furthermore, our thorough analysis of case–control studies revealed a significant correlation between *miR‐542‐3p* and HCC, as evident from the sensitivity and specificity values obtained. Network analysis was performed to determine the relationship between *miR‐542‐3p* and its function in the liver.

Tao et al. observed a significant decrease in *miR‐542‐3p* levels in HCC tissue and cell line samples. In 2016, Rang et al. revealed an interesting finding regarding the association between *miR‐542‐3p* and the proto‐oncogene PIM1 in melanoma [10]. Preclinical analysis showed that decreased *miR‐542‐3p* expression resulted in increased cancer cell migration, spreading, and epithelial–mesenchymal transition (EMT). However, the HCC phenotype, characterised by aggressiveness and recurrence, exhibited reduced *miR‐542‐3p* expression. Reduced *miR‐542‐3p* expression correlates with aggressive clinicopathological characteristics in individuals with HCC, such as advanced tumour, node, and metastasis (TNM) stage and venous infiltration [15].

Nevertheless, our study, which is the first meta‐analysis of *miR‐542‐3p*, provides strong evidence for the predictive significance of *miR‐542‐3p* expression in HCC patients. Studies by Tao et al. and Tu et al. revealed significant findings regarding the effect of *miR‐542‐3p* on the migration and spread of cancerous cells. Their studies demonstrated that overexpression of *miR‐542‐3p* effectively suppressed these metastatic characteristics, whereas knockout of *miR‐542‐3p* had an inverse effect, enhancing the migration and invasion of HCC cells, and knockout of *miR‐542‐3p* enhanced these metastatic characteristics through functional gain and loss. According to previous studies, *miR‐542‐3p* expression inhibits HCC [15, 16]. In accordance with the research conducted by Tao et al., our results also support the significant function of *miR‐542‐3p* as a suppressor gene and hold significant potential as a target for the clinical therapy of alcohol‐mediated HCC.

Furthermore, these data indicate that *miR‐542‐3p* has the potential to be utilised not only for diagnosis but also for tracking treatment response and disease progression in patients with HCC. Further studies should be conducted with a larger number of datasets to observe the impact of the biological activity of *miR‐542‐3p* in continuous alcoholics and those at high risk of developing alcohol‐mediated HCC. Our study had some limitations. After reviewing the data sources between 2021 and 2024 by meta‐analysis, the authors found that there are limited data or sources regarding the role of *miR‐542‐3p* in HCC. However, a randomised cohort study specifically targeted a population of South Asian adults aged 40–50 years. These results may not be generalisable to other populations or age groups. Nevertheless, the authors believe that larger sample sizes (datasets) would provide more robust and reliable conclusions.

### 4.1 Mechanism of *miR‐542‐3p* in Cancer Progression

**FIGURE 4:**
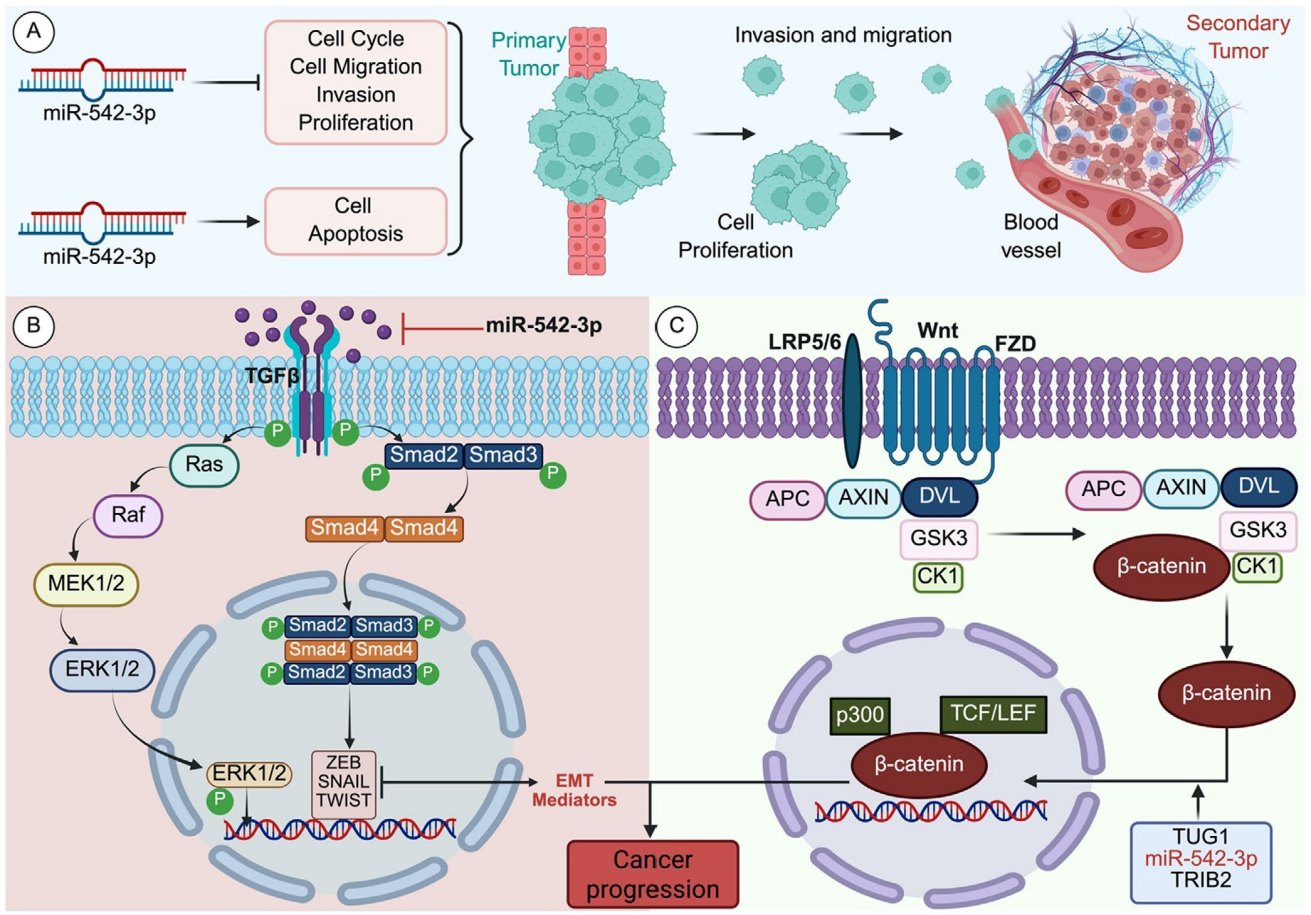
(A‐C) *miR‐542‐3p* modulates tumour progression by influencing the cell cycle, migration, apoptosis, and invasion via the TGF‐β/Smad and Wnt/β‐catenin signalling pathways, contributing to cancer metastasis and proliferation.

This study examined the role of *miR‐542‐3p* in cancer progression, specifically its effects on cell proliferation, migration, invasion, apoptosis, and metastasis. The mechanism is categorised into three distinct sections (A, B, and C), each depicting various signalling pathways and their association with *miR‐542‐3p* (Figure 4).

#### 4.1.1 *miR‐542‐3p* Role in Primary Tumour and Metastasis

Section (A) (Figure 4) illustrates the function of *miR‐542‐3p* in regulating multiple cellular processes associated with tumour progression. The impact of *miR‐542‐3p* was observed in the regulation of the cell cycle, as well as in processes such as migration, invasion, and proliferation. These functions facilitate the advancement of primary tumours and their progression to metastasis. This is illustrated by the invasion of tumour cells into adjacent tissues and their migration through blood vessels, leading to the establishment of secondary tumours at distant locations [24]. *miR‐542‐3p* induces apoptosis, a form of programmed cell death, thereby inhibiting cancer progression. This illustrates the dual function that *miR‐542‐3p* can exhibit, either by suppressing or facilitating tumour growth, depending on the specific cellular environment [25]. This panel emphasises the intricate equilibrium between the pro‐apoptotic and pro‐proliferative functions of *miR‐542‐3p*, which plays a crucial role in determining cancer cell fate.

#### 4.1.2 TGF‐β Pathway and *miR‐542‐3p* in EMT

In section (B) (Figure 4), the Transforming Growth Factor Beta (TGF‐β) signalling pathway is a significant regulator of epithelial‐mesenchymal transition (EMT), a vital process in cancer metastasis [26]. The essential steps include activation of the TGF‐β receptor; when TGF‐β binds to its receptor, the Smad‐dependent pathway is activated. Smad protein phosphorylation occurs when the receptor phosphorylates Smad2 and Smad3. These phosphorylated proteins subsequently associate with Smad4 to form a complex [27]. Nuclear translocation occurs when the SMAD complex moves into the nucleus, influencing the expression of EMT genes, such as ZEB, SNAIL, and TWIST. The transcription factors in question repress markers associated with epithelial cells while simultaneously activating those linked to mesenchymal cells. This process facilitates epithelial‐mesenchymal transition (EMT), allowing tumour cells to migrate, invade, and exhibit resistance to programmed cell death. The function of *miR‐542‐3p* is to inhibit the targeting components involved in TGF‐β signalling, which may obstruct the transition from primary tumour to metastatic spread. By inhibiting the TGF‐β/Smad pathway, *miR‐542‐3p* restricts cancer cell invasion and migration, thereby blocking aggressive dissemination [24].

#### 4.1.3 Wnt/β‐Catenin Pathway and Cancer Progression

Section (C) (Figure 4) illustrates the Wnt/β‐catenin signalling pathway, which is crucial for cancer development and metastasis [28]. The significant occurrence of this pathway encompasses the interaction of Wnt proteins with the receptors LRP5/6 and FZD (Frizzled), resulting in the suppression of the APC/AXIN/GSK3 complex, which typically facilitates the degradation of β‐catenin. Stabilisation of β‐catenin: β‐catenin accumulates in the cytoplasm in the absence of degradation and is subsequently translocated to the nucleus. Nuclear signalling involves the formation of a complex between β‐catenin and transcription factors, such as TCF/LEF, within the nucleus, leading to the activation of target genes that play roles in cell proliferation and cancer progression [29]. *miR‐542‐3p*, in conjunction with other molecules such as TUG1 and TRIB2, affects β‐catenin activity. Through its regulatory activity, *miR‐542‐3p* may influence β‐catenin levels, thereby affecting cancer progression. In this instance, *miR‐542‐3p* appears to function as a tumour suppressor by obstructing elements that promote β‐catenin‐mediated transcriptional activity, thereby averting the unregulated proliferation and invasion of cancer cells [24, 30].

#### 4.1.4 Integration of Pathways and Cancer Progression

The combined effects of the TGF‐β/Smad and Wnt/β‐catenin pathways drive epithelial‐mesenchymal transition (EMT), a key mechanism of cancer metastasis [31]. EMT facilitates the acquisition of mesenchymal characteristics by cancer cells, thereby enhancing motility and invasiveness. Figure 4 demonstrates the role of these pathways in cancer progression by inducing alterations in the cellular phenotype that aid in the dissemination of tumours to secondary locations. EMT mediators, such as ZEB, SNAIL, and TWIST, play crucial roles in regulating genes associated with cellular adhesion, cytoskeletal organisation, and motility. These molecules facilitate the transition of epithelial cells to mesenchymal cells, which exhibit increased invasiveness and resistance to apoptosis [32]. Cancer progression involves the acquisition of mesenchymal characteristics by tumour cells, which enables them to infiltrate adjacent tissues, access the bloodstream, and establish secondary tumours in distant organs. The regulation of these processes by *miR‐542‐3p* highlights its significance in controlling cancer metastasis [24].

## 5 Conclusion

In summary, despite certain limitations, our findings suggest that miR‐542‐3p may serve as a promising molecular target in alcohol‐associated HCC, particularly in moderate drinkers, highlighting its potential role in future therapeutic strategies. The role of *miR‐542‐3p* as a marker for cancer therapy and prognosis can assist in identifying individuals who are more susceptible to cancer progression. We performed a meta‐analysis to demonstrate the robustness of our study, and the analysis supported the importance of *miR‐542‐3p* in HCC development. *miR‐542‐3p* is critical for regulating cancer progression by targeting the TGF‐β/Smad and Wnt/β‐catenin signalling pathways. Through these interactions, *miR‐542‐3p* can influence EMT, invasion, migration, and metastasis, making it a potential therapeutic target for preventing cancer spread. Further studies with larger sample sizes are recommended to enhance our understanding of the correlation between *miR‐542‐3p* and HCC.

## Author Contributions

**Ranjith Balakrishnan:** conceptualization (equal), data curation (equal), formal analysis (equal), investigation (equal), methodology (equal), resources (equal), software (equal), validation (equal), writing – original draft (equal). **Rajasekaran Subbarayan:** conceptualization (equal), data curation (equal), formal analysis (equal), investigation (equal), methodology (equal), project administration (equal), resources (equal), software (equal), supervision (equal), validation (equal), writing – original draft (equal). **Maheshkumar Kuppusamy:** conceptualization (equal), formal analysis (equal), methodology (equal), project administration (equal), supervision (equal), validation (equal), writing – review and editing (equal). **Rupendra Shrestha:** conceptualization (equal), formal analysis (equal), investigation (equal), methodology (equal), project administration (equal), resources (equal), visualization (equal), writing – review and editing (equal). **Arunkumar Radhakrishnan:** formal analysis (equal), investigation (equal), methodology (equal), validation (equal), visualization (equal), writing – review and editing (equal). **Ankush Chauhan:** formal analysis (equal), resources (equal), validation (equal), visualization (equal), writing – review and editing (equal).

## Ethics Statement

The authors have nothing to report.

## Consent

The authors have nothing to report.

## Conflicts of Interest

The authors declare no conflicts of interest.

## Supporting information


Data S1.

## Data Availability

The data that supports the findings of this study are available in the Supporting Information of this article.
