# Supplementary material for: Circulating miR‐542‐3p as a Prognostic Marker for Hepatocellular Carcinoma: A Systematic Review and Meta‐Analysis
Source: J Cell Mol Med. 2025 Jul 27;29(14):e70748. doi: 10.1111/jcmm.70748 (PMC12301263; doi:10.1111/jcmm.70748)
Supplement: Supplementary file 1 — Data S1. [file JCMM-29-e70748-s001.docx]

**Supplementary Data**

**Supplementary Figure and Legends**

**
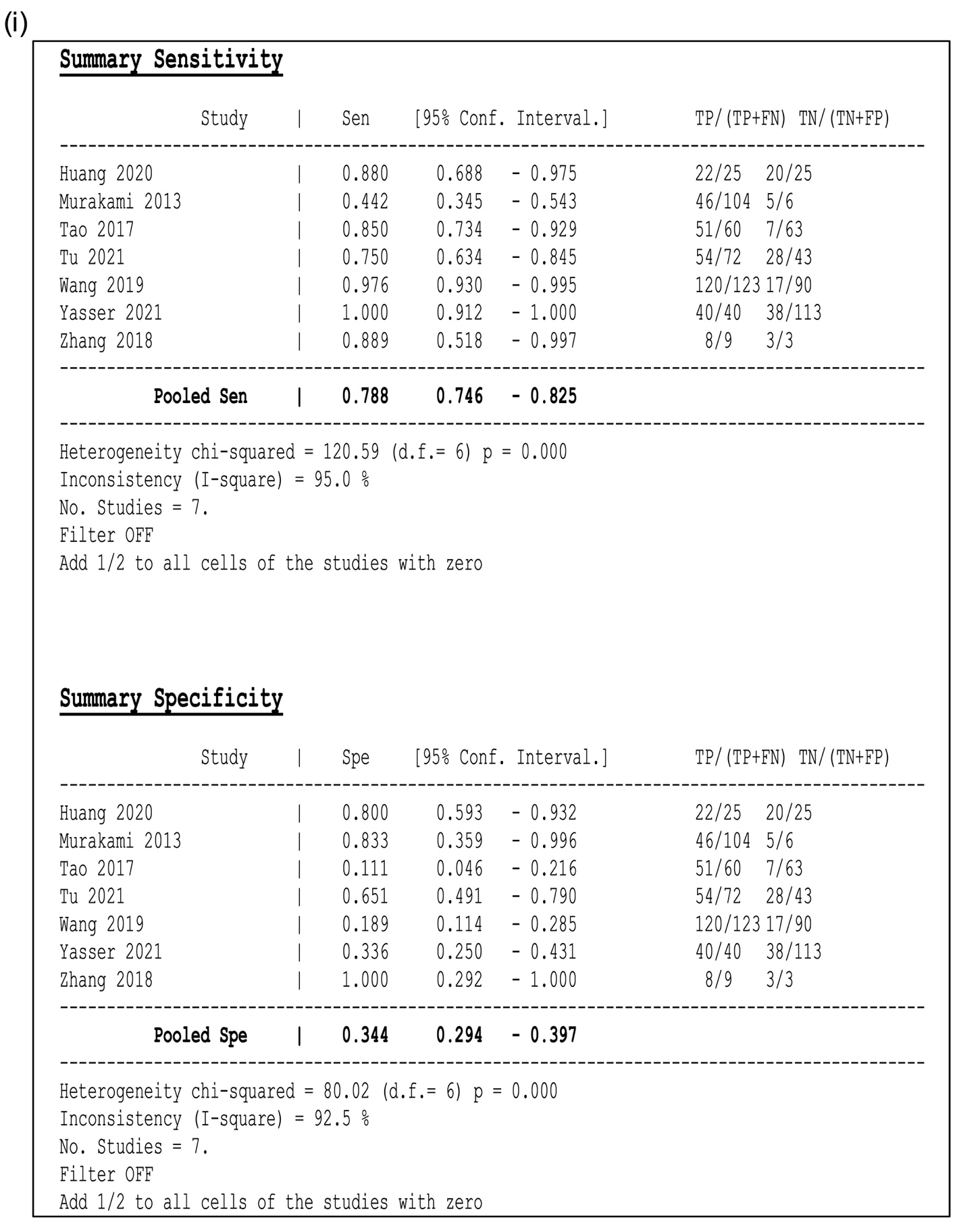
**

**
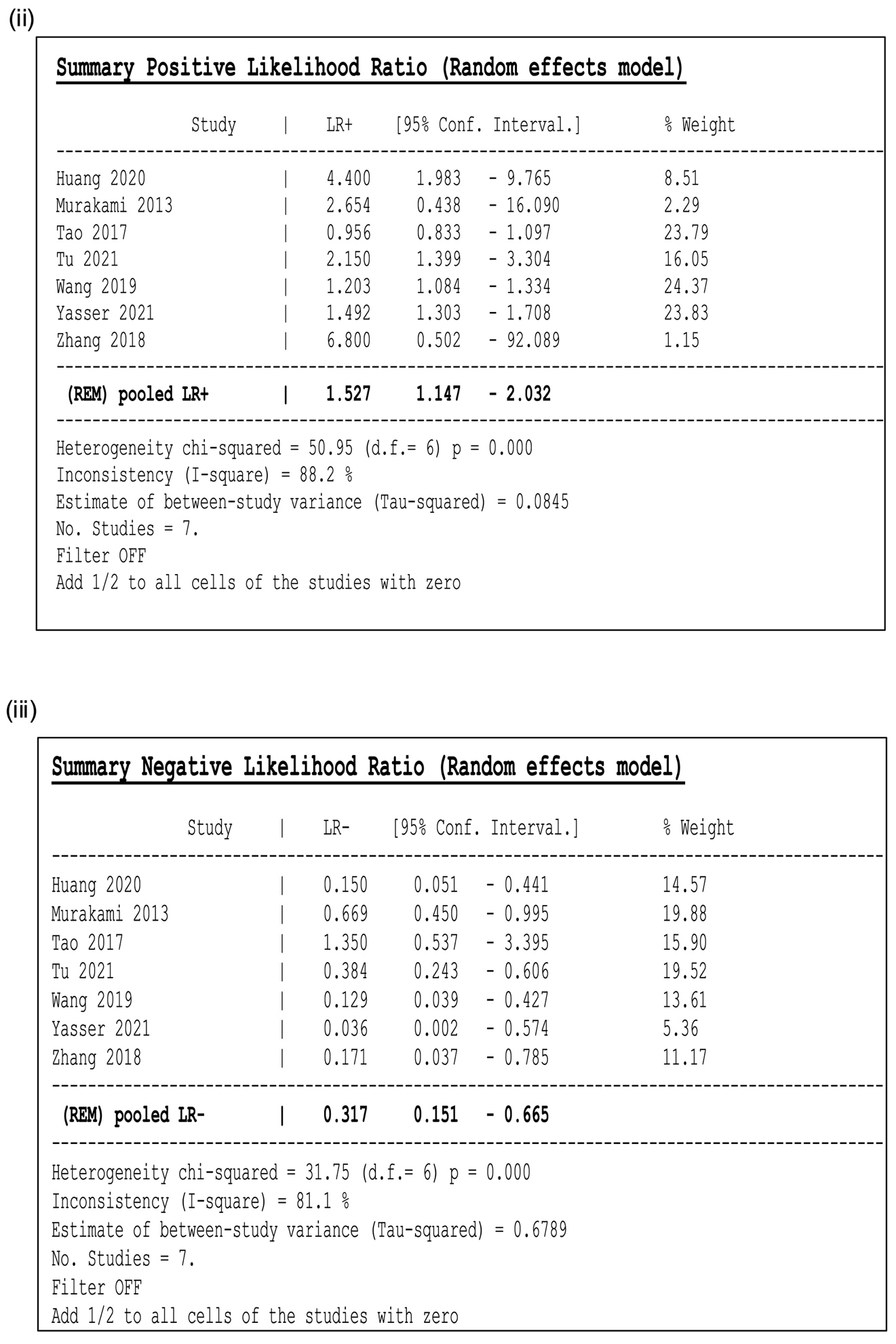
**

**
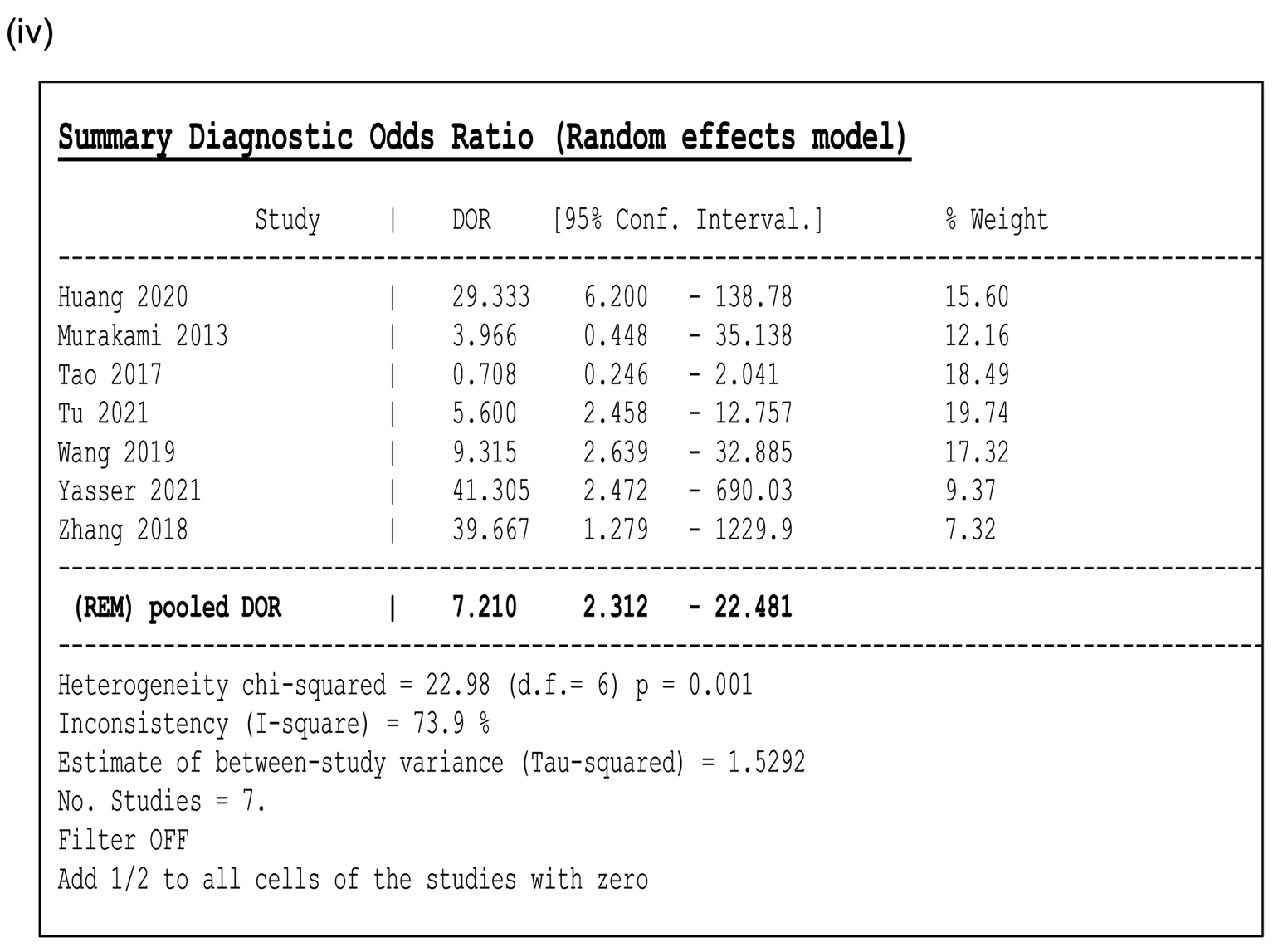
**

**Supplementary Figure 1:** Pooled analysis calculating the sensitivity (i), specificity (ii), (iii) PLR, NLR, and (iv) DOR for the included studies.


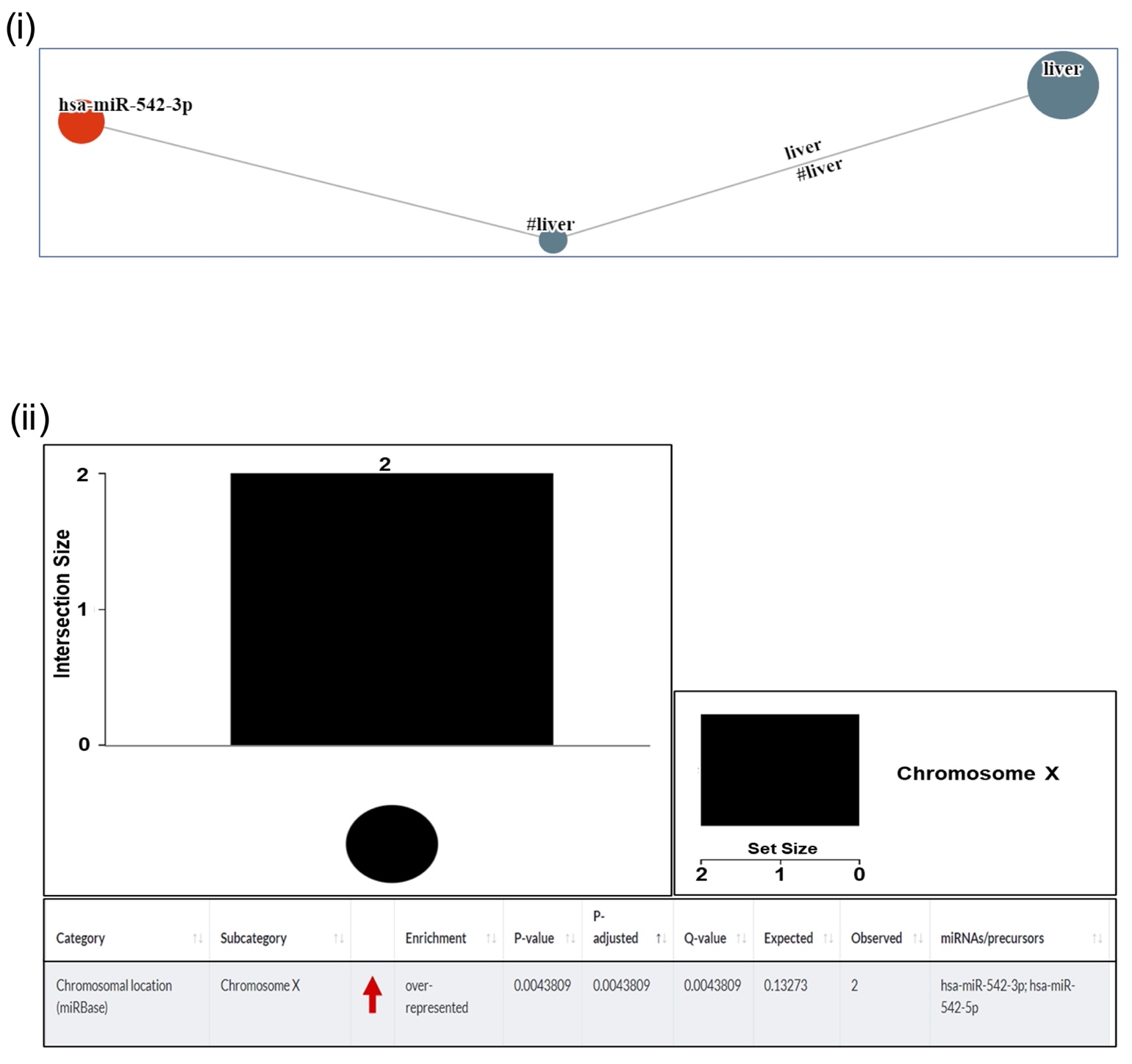


**Supplementary Figure 2:** (i) Network analysis of miR-542-3p obtained from the tissue atlas database. (ii) Depicts the chromosomal location of miR-542-3p (Obtained using ‘miRBase’ database: <http://www.mirbase.org/>).

**Supplementary Table**

**Supplementary Table 1:** Important aspects and degrees of methodological coherence of the eligible studies. [PB: Population-based, HB: Hospital-based].

| **Author** | **Year** | **Country** | **Continent** | **Ethnicity** | **Genotyping method** | **Sample** | **Case/**  **Control** | **Source of control (PB or HB)** |
| --- | --- | --- | --- | --- | --- | --- | --- | --- |
| **Huang et al. 2020** | 2020 | China | Asia | Asian | RT-PCR | Tissue | 25/25 | HB |
| **Murakami et al. 2013** | 2013 | Japan | Asia | Asian | qRT-PCR | Tissue | 104/6 | HB |
| **Tao et al. 2017** | 2017 | China | Asia | Asian | RT-PCR | Tissue | 123/123 | HB |
| **Tu et al. 2021** | 2021 | China | Asia | Asian | qRT-PCR | Tissue | 115/115 | HB |
| **Wang et al. 2019** | 2019 | China | Asia | Asian | qRT-PCR | Tissue | 213/213 | HB |
| **Yasser et al. 2021** | 2021 | Egypt | Africa | Caucasian | qRT-PCR | Blood | 115/38 | HB |
| **Zhang et al. 2018** | 2018 | China | Asia | Asian | qRT-PCR | Tissue | 9/3 | HB |
